# Perceptions of hypnotherapy for children with functional abdominal pain: a qualitative study

**DOI:** 10.1093/fampra/cmaf066

**Published:** 2025-08-26

**Authors:** Ilse N Ganzevoort, Adriëlla L Van der Veen, Manna A Alma, Anja Karg, Marjolein Y Berger, Gea A Holtman

**Affiliations:** Department of Primary and Long-Term Care, University of Groningen, University Medical Center Groningen, Groningen, The Netherlands; Department of Primary and Long-Term Care, University of Groningen, University Medical Center Groningen, Groningen, The Netherlands; Department of Health Sciences, Applied Health Sciences, University of Groningen, University Medical Center Groningen, Groningen, The Netherlands; Department of Primary and Long-Term Care, University of Groningen, University Medical Center Groningen, Groningen, The Netherlands; Department of Primary and Long-Term Care, University of Groningen, University Medical Center Groningen, Groningen, The Netherlands; Department of Primary and Long-Term Care, University of Groningen, University Medical Center Groningen, Groningen, The Netherlands

**Keywords:** primary health care, abdominal pain, child, adolescent, hypnosis, qualitative research

## Abstract

**Background:**

Hypnotherapy is an effective treatment for children with functional abdominal pain in secondary care. However, children usually present first to general practitioners (GPs) in Dutch primary care, and it is unknown how children, parents, and GPs perceive hypnotherapy in this setting.

**Objective:**

To explore the perceptions of children, parents, and GPs about hypnotherapy in primary care for children with functional abdominal pain.

**Methods:**

This is a qualitative study among Dutch children with functional abdominal pain, the parents of other children with functional abdominal pain, and GPs. Semi-structured interviews were conducted online. Interviews were recorded and transcribed verbatim. Data were analysed iteratively by thematic content analysis.

**Results:**

In total, 7 children, 8 parents, and 12 GPs participated. We identified three main themes: attitude to abdominal pain, therapeutic context, and societal constraints and considerations. The attitudes of children and parents to abdominal pain, including acceptance of pain as functional and coping behaviour, affected their expectations of hypnotherapy. Therapeutic context (e.g. the GP–patient relationship and expectations of an effect) and societal constraints and considerations (e.g. stigma and cost) regarding hypnotherapy affected the willingness of children, parents, and GPs to use hypnotherapy.

**Conclusions:**

Despite controversy about its use, hypnotherapy for functional abdominal pain is considered as a treatment option in primary care. Attitudes of children and parents influenced their willingness to use hypnotherapy. Effective implementation requires good information about hypnotherapy, a good GP–patient relationship, and clear and reliable referral options for hypnotherapy.

Key messages

- Hypnotherapy for treating abdominal pain in children is considered in primary care.
- Attitude to abdominal pain, therapeutic, and societal context influence perceptions.
- Controversy exists among general practitioners (GPs) on its use and applications.
- For implementation, GPs require information on hypnotherapy and referral options.

## Introduction

Functional abdominal pain disorders in children are common. Symptoms are characterized by recurrent abdominal pain for more than 2 months and cannot be explained by another medical condition after appropriate medical evaluation [1]. Hypnotherapy (medical hypnosis) has emerged as a treatment for children with functional abdominal pain [2]. It is used to induce a trance state in which a person's concentration increases, allowing the acceptance of suggestions given by a therapist to induce changes in physiology, sensation, emotion, thought, or behaviour [3, 4]. Despite low certainty of evidence, two independent systematic reviews concluded that hypnotherapy reduces abdominal pain in children and adolescents and that hypnotherapy may be considered for treating functional abdominal pain in secondary care [5, 6]. In addition, home-based guided hypnotherapy, where a patient listens to recorded audio exercises online, is non-inferior to face-to-face hypnotherapy with a therapist at a lower cost of only 30 euros and requires less time investment [7]. Given that hypnotherapy is an active treatment that requires an investment of time and energy, implementation could be hampered without acceptance from the children and parents who must adopt the practice, and without the willingness of healthcare professionals to offer it as a treatment.

The perception of hypnotherapy has been reported in adults. Almost half of the general adult population have neutral perceptions, almost 40% have positive perceptions, and >10% would reject or not seek the treatment [8, 9]. Adult patients with irritable bowel syndrome also report several barriers, including time, costs, fears and misconceptions, lack of awareness, and insufficient knowledge about its effects [10, 11]. A study in the USA found that mothers and children aged 7–12 years with functional abdominal disorders held positive views about using an app for therapy based on guided imagery [12]. However, the perceptions of hypnotherapy among children with functional abdominal pain and their parents are unknown.

In Dutch primary care, children with functional abdominal pain usually first see a general practitioner (GP), who provides education and reassurance [13]. Evidence-based treatments are lacking when symptoms persist. Although paediatricians use hypnotherapy by referring to qualified therapists, it is not offered by GPs due to insufficient evidence for effectiveness in this setting. Introducing hypnotherapy in primary care could offer an accessible, cost-effective option that may prevent symptom progression and referrals [14]. Understanding perceptions of hypnotherapy is a key to supporting its implementation. Therefore, this study aimed to investigate the perceptions of hypnotherapy in primary care for children with functional abdominal pain from the perspectives of affected children, parents, and GPs.

## Methods

We conducted a qualitative study using semi-structured interviews with children, parents, and GPs. Reporting follows the Consolidated Criteria for Reporting Qualitative Research (COREQ) checklist [15]. The Medical Ethics Review Committee of the University Medical Centre Groningen, The Netherlands, confirmed that the Medical Research Involving Human Subjects Act (WMO), which includes consideration of the Declaration of Helsinki, did not apply to our study (number 202200110). All participants gave informed consent before each interview.

### Participants

We included children aged 12–18 years and the parents of children aged 7–14 years, from different families, who visited their GP to manage functional abdominal pain, only if they spoke Dutch. Participants were approached by telephone after giving contact information for potential participation in an ongoing randomized controlled trial evaluating the (cost-)effectiveness of hypnotherapy in primary care [16]. At the time of the interviews, none of the children had been randomized or received hypnotherapy. Purposive sampling was used to strive for variety in age, sex, and parental education level for the children and parents.

GPs (including trainees) were eligible if they worked in the Netherlands. We invited GPs who participated or refused to participate in the trial and through networking. Purposive sampling was used to strive for variety in sex, years working as a GP, and awareness of hypnotherapy (already refers to, is likely to use, or is against its use).

### Data collection

We collected data by in-depth semi-structured interviews with children, parents, and GPs between July 2022 and June 2023. Based on existing literature and expert discussion, an interview guide was developed, and the language was adjusted for use with each group. The guide comprised open questions about abdominal pain (children and parents only); knowledge, expectations, and intrinsic motivation concerning hypnotherapy; and applicability of hypnotherapy in primary care (Supplementary 1). After five interviews, the interview guide was evaluated in a research team discussion, and adjustments were made as needed.

Online interviews (via Microsoft Teams) were conducted in Dutch by a female primary care researcher and trained interviewer (I.N.G.), who presented herself as a researcher aiming to study perceptions of hypnotherapy. Interviews were video and audio recorded before being transcribed verbatim; field notes were made after each interview to provide context. Data collection was planned to stop after reaching data saturation (i.e. interviews no longer generated relevant concepts). Member check was performed by emailing all participants a summary of their interview.

### Data analysis

Data were collected and analysed iteratively, allowing emerging themes to be incorporated and explored in subsequent interviews. Thematic content analysis, as proposed by Braun and Clarke [17], was used to analyse the data in ATLAS.ti (version 23). Two researchers (I.N.G. and A.L.V.d.V.) independently read and coded the first five transcripts and generated a draft codebook that was adapted for coding. The remaining transcripts were coded by I.N.G. and checked by A.L.V.d.V. Finally, codes were clustered into themes and subthemes and discussed during research team meetings with experts from different backgrounds, and any inconsistencies were resolved until consensus was reached (Supplementary 2). Quotes were translated from Dutch to English by the first author, edited by a native English speaker, and then rechecked by the first author to ensure that their intended meaning was retained.

## Results

### Participants

Of 44 invited participants, 17 were not interested because of the time investment (*n* = 2), personal reasons (*n* = 1), pain resolution (*n* = 1), or no reason (*n* = 13). Data saturation appeared after 21 interviews, and we have added another five interviews to confirm saturation. Variation in all purposive sampling characteristics was achieved. We conducted 26 semi-structured interviews with 27 participants: 7 children, 8 parents, and 12 GPs, each lasting 17–74 min (Table 1). One interview included two GPs and is counted as a single interview. All participants agreed with the summaries of their interview content.

**Table 1.** Participant characteristics.

Characteristic	Children, *n* (*n* = 7)	Parents, *n* (*n* = 8)^a^	GPs, *n* (*n* = 12)
Gender, female	4	6	8
Age, years			
12	1		
13	3		
16	2		
18	1		
25–30		3	1
31–40		4	2
41–50		1	5
51+			3
Educational level, mother			
Intermediate	2		
High	2		
Unknown	3		
Educational level, father			
Intermediate	3		
High	1		
Unknown	2		
NA, single-parent family	1		
Educational level^b^			
Intermediate		2	
High		6	
Work as GP, years			
In training			1
1–10			6
11–20			3
21+			2
Hypnotherapy predisposition			
Refers to			3
Likely to use			8
Against use			1
Trial participation, yes	6	5	7

GP, general practitioner; NA, not applicable.

^a^Five female and three male children, median age 9.5 years (range: 8–14).

^b^Partners had similar educational levels to the interviewed parent. Educational level was considered low (primary and lower secondary), intermediate (secondary vocational), or high (bachelor's degree or higher) [18, 19].

### Themes

**Figure 1.**
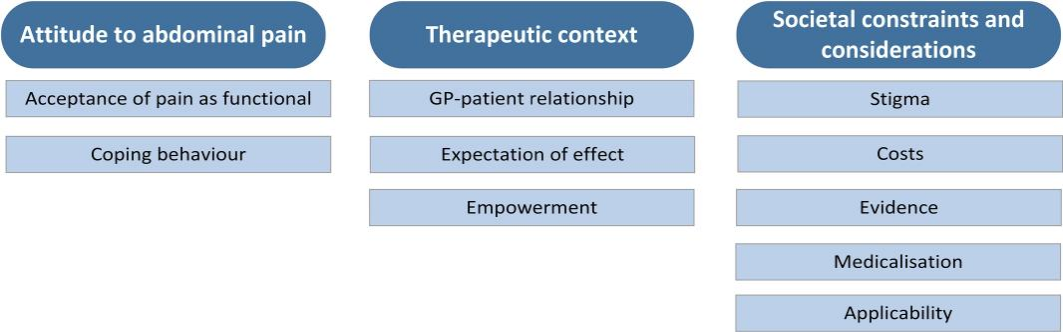
Main themes and subthemes. Abbreviation: GP, general practitioner.

We identified three main themes concerning the perceptions of hypnotherapy in primary care: attitude to abdominal pain, therapeutic context, and societal constraints and considerations. The attitudes of children and parents to abdominal pain influenced how willing they were to try hypnotherapy. Therapeutic context and societal constraints and considerations influenced whether children, parents, or GPs accepted hypnotherapy as a primary care treatment. Figure 1 summarizes the main themes and subthemes.

### Attitude to abdominal pain

Attitudes to abdominal pain included the acceptance of pain as being functional in nature and associated coping behaviours.

#### Acceptance of pain as functional

Children and parents noted that a reassuring explanation given by the GP, or a normal test result, helped them accept the pain as functional. This reassurance made them more open to trying hypnotherapy.

*I want to make sure that nothing is wrong. […] But after that, if lab results are good, etc., the time is right [for GPs] to offer hypnotherapy, because it gives someone a tool to do something. (Parent 6, female, age 44 years)*

Other children and parents did not entirely accept that the abdominal pain was functional. Although some agreed that the pain may be triggered by stress or other factors, they feared a severe underlying pathology and wanted further examination. However, this group was still willing to try hypnotherapy because it might help or because they were ‘a little desperate’ (Child 7, female, age 16 years).

GPs also emphasized the importance of children and parents accepting the pain and other unexplained symptoms (e.g. headache and nausea) as functional, citing that worries about the cause of symptoms could maintain them. Several GPs expected a better response to hypnotherapy if parents and children accepted the pain as functional.

*[Offering hypnotherapy] is only possible when children and parents think, ‘we are reassured, nothing serious is going on; but, we want to get rid of it’. If you are at that point, I think they will be open [to try hypnotherapy], but if you offer it too early, you might trivialise the problem. (GP 2, male, age 52 years, 5.5 years' experience)*

#### Coping behaviour

Most children and parents wanted help finding a coping strategy that could reduce or remove the pain, and although some had more actively searched for strategies than others, most welcomed hypnotherapy. Some did not know how to deal with the pain and wanted a solution; others had become used to the pain, tried many different coping strategies, and found their own strategies.

*Because you have had [abdominal pain] for a long time, we know how to work around it. (Parent 3, female, age 37 years)*

Some children and parents wanted to find ways to deal with the pain, consistent with statements by GPs. Coping included trying to find comfort, changing diets, or using breathing exercises. Distraction was an important strategy: school and playing with friends worked well during the day, whereas gaming, listening to music, or reading worked at other times. However, if they visit their GP, parents and children expect to be offered new coping strategies. They might include hypnotherapy.

*The GP might have a solution [to reduce pain]; I am willing to try, and if it works, I will be happy. (Child 3, female, age 13 years)*

Some parents revealed that, if the GP could not offer tools to help their child deal with the pain, they wanted a referral to someone who could.

### Therapeutic context

Therapeutic context affects the expectations of hypnotherapy among children, parents, and GPs. We identified three subthemes: GP–patient relationship, expectation of effect, and empowerment.

#### GP–patient relationship

GPs mentioned a lack of treatment options for these patients and suggested that hypnotherapy could be a welcome tool. They emphasized that offering a treatment that could work helps maintain or build a stronger relationship with the patient.

*If you say, ‘we are going to do this’, and it helps, then people will think: ‘see! My doctor was right and helped me to get rid of my symptoms’, then they are more inclined to follow your advice next time. (GP 3, female, age 37 years, 9 years’ experience)*

Most children and parents mentioned the need for a trusting relationship with their GP to consider new treatment options, such as hypnotherapy, if mentioned. Parent 6 (female, age 44 years) reported that this would ‘offer a perspective’.

GPs acknowledged the need for a good patient–doctor relationship and for them to have faith in hypnotherapy when recommending it for treatment. Another precondition was that GPs should be able to explain hypnotherapy to both the child and their parents.

*If you are convinced about the treatment yourself, then you can easily convince most people that it is good for them. People with whom you have a good relationship, or you really take all steps… most will go along with that. (GP 1, male, age 59 years, 25 years’ experience)*

#### Expectation of effect

The expectations of hypnotherapy among children, parents, and GPs affected their motivation for treatment. Participants were more willing to try hypnotherapy if they expected pain reduction or improvements in mood, relaxation, or positive outlooks.

*If [hypnotherapy] offers the chance for many of my symptoms to go away … then I can just live my life more comfortably. If it works well, I expect it will make a big change to my life. (Child 2, male, age 16 years)*

We also found cues that expectations can be influenced by positive experiences with similar therapies.

*She is really open for [hypnotherapy]…that is also because of her experience with the worry balloon, which helps her. (Parent 5, female, age 42 years)*

Some parents and GPs expected better compliance, and therefore effectiveness, with face-to-face hypnosis. Even without knowing that hypnotherapy will help, most children and parents would try it because they have nothing to lose. Many participants noted time, money, and logistics as possible disadvantages. However, they considered the advantages to be more important, including a lack of harmful or negative side effects, positivity about hypnotherapy not being a medicine, and the expectation of a longer beneficial effect.

*[Hypnotherapy] can only bring benefit; you cannot do any harm with this, so it can only be good. (Parent 7, female, age 38 years)*

Some GPs expected no effect, or if one occurred, would interpret this as a placebo effect. These GPs were more hesitant to offer hypnotherapy or would not offer hypnotherapy at all.

*Placebo might help, but I would rather see another solution for these children. (GP 12, male, 42 years, 12 years' experience)*

#### Empowerment

Most participants considered hypnotherapy a tool that empowers children, giving them control over their body, the pain, and coping with situations. GPs revealed that they wanted to give their patients a sense of self-reliance, which hypnotherapy could offer. Parents and GPs both emphasized the importance of empowering children with the necessary tools to cope well in adulthood.

*If you get to know that you can make yourself better, or influence yourself, that not only gives a nice feeling, because you are in control, but also provides a useful skill that might come in handy. (GP 10, female, age 45 years, 10 years' experience)*

All participants agreed that hypnotherapy requires time and effort, and several mentioned the need for motivation. A child's busy schedule with school, sports, and other commitments could make adherence difficult. Consequently, home-based guided hypnotherapy was considered more accessible and convenient. Children noted being motivated to comply with hypnotherapy to manage their pain.

*I think I could always find a way to trade half an hour or 20 minutes, for example, of an episode of a series [to do hypnotherapy]. (Child 1, male, age 18 years)*

### Societal constraints and considerations

We identified five subthemes of societal constraints and considerations that affect the perception of hypnotherapy: stigma, costs, evidence, medicalization, and applicability.

#### Stigma

When first thinking of hypnotherapy, most participants mentioned that they imagined television shows or movies. It was viewed to be a magic trick in which someone takes control of your body while you do not know what is happening. Some participants mentioned that the term hypnotherapy might be replaced to avoid stigma. GPs expect resistance from patients if they mention hypnotherapy.

*I think that [patients] would think I was really foolish at first. Especially because hypnotherapy—I would not want to call it taboo—it has a certain [negative] charge in society. (GP 4, female, age 27 years, in training)*

For some, the stigma disappeared after reading or hearing information about hypnotherapy. Others did not stigmatize hypnotherapy from the start because they already knew about, or had experience with, hypnotherapy or comparable therapies (e.g. mindfulness).

*Everything with ‘hypno-’ sounds a bit vague. Well, that was what I thought of mindfulness as well, until you read where it comes from, and you start doing it yourself. (Parent 8, male, age 45 years)*

#### Costs

Many participants mentioned that costs could be a burden when engaging in hypnotherapy. GPs would find it difficult to offer hypnotherapy to patients if a payment was needed. Without reimbursement by insurance companies, GPs and parents thought that access based on ability to pay would be inequitable.

*It is a chronic problem. It is not something that … will ever go away. Therefore, I think it should be [covered by] some sort of standard insurance. (Parent 3, female, age 37 years)*

Other GPs mentioned that insurance should not cover hypnotherapy because it is an unproven placebo therapy, or because higher costs would make the therapy more serious and could therefore yield a greater effect. However, some commented that you must weigh the pros and cons.

*The disadvantage of hypnotherapy [high costs] is more important than the advantage of possible improved compliance. (GP 2, male, age 52 years, 5.5 years' experience)*

#### Evidence

Many GPs emphasized the importance of evidence-based therapies. With proven effectiveness in primary care, most GPs would embed hypnotherapy in their treatment.

*If there is evidence, you can just use it. (GP 1, male, age 59 years, 25 years' experience)*

We encountered resistance from some GPs who mentioned that double-blind studies could not be performed to prove its effectiveness, instead considering positive outcomes to be placebo effects that deceive patients.

*It is very difficult to come up with a study design, because you cannot test double-blind, randomised. So, there will always be placebo and nocebo effects. And then it will probably come out that … this helps very well, yes. (GP 12, male, 42 years, 12 years' experience)*

Supporting evidence would help children and parents decide whether to start hypnotherapy.

*For me, it would be helpful if [the GP] said something about the evidence. Not that it has to be very data driven, but to explain that good results have been achieved. (Parent 6, female, age 44 years)*

#### Medicalization

Several participants mentioned that they did not consider further investigations, medications, or hospital referrals necessary. Such medicalization would only magnify the pain, and most GPs wanted it to be normalized. Some GPs mentioned that introducing hypnotherapy early could magnify the pain's importance.

*I would like to be careful not to make it a problem. Please, let's not send all children with functional abdominal pain to a hypnotherapist. (GP 9, female, age 34 years, 3 years' experience)*

Other GPs mentioned that hypnotherapy may decrease care demand by helping patients better understand their body and find ways to deal with their pain instead of visiting a healthcare professional.

*You also protect your patients from unnecessary medical intervention, which is important to reduce pressures on medical care and to ensure that patients only go to secondary care when needed. (GP 3, female, age 37 years, 9 years' experience)*

#### Applicability

GPs mentioned seeing small numbers of patients with functional abdominal pain. In most cases, they reported that education and reassurance were sufficient. Given that few of these patients return, hypnotherapy would only be applicable to a small number of children.

*Regarding whether you could offer hypnotherapy as a first-line intervention, I think, sure. But, the group that really needs it might be quite small. (GP 5, female, age 37 years, 6 years' experience)*

Despite this, participants stated that hypnotherapy could be used to help children and adults with other symptoms, such as headache, sleep issues, and chronic pain.

Most GPs wanted to refer patients to a therapist or to home-based guided hypnotherapy. Some parents and GPs emphasized that a therapist should be appropriately qualified. GPs mentioned the need for training and for publicity in GP journals. They wanted this to describe hypnotherapy, what it entails, and how to convey this information to patients. They also wanted information about referral options.

*I would not know how I refer, or how I explain what it is. So no, I cannot do anything with that yet. (GP 7, female, age 50 years, 20 years' experience)*

Some GPs wanted training to deliver hypnotherapy themselves if there would be sufficient time and money, but others did not share this desire.

## Discussion

### Strengths and limitations

A strength of this study is the data source triangulation [20]. We included both the patient perspective, by interviewing children and parents, and the practitioner perspective, by interviewing GPs. Another strength is that we had a diverse research team comprising researchers, an epidemiologist, a social scientist with expertise in qualitative research, and GPs for discussing and analysing any codes and themes. Viewing data from these different perspectives improved the analysis.

A limitation is that we primarily interviewed children and parents who were recruited after showing interest in hypnotherapy in a trial. However, we heard negative and neutral opinions from children and parents and included one GP with negative perceptions of hypnotherapy. Additionally, because we only included participants with a Dutch cultural background, those with other cultural backgrounds could have different perceptions [21].

### Comparison with existing literature

Our study confirmed the need for clear communication that the pain is functional for acceptance, reassurance, and managing the pain [22–26]. Most children and parents would like to use hypnotherapy as a coping strategy, consistent with another study reporting that many children and parents already use similar coping strategies, such as distraction, meditation, and breathing exercises [27, 28].

Our study adds to the existing evidence of controversy and stigma among adults, children, parents, and GPs surrounding hypnotherapy [11, 29, 30]. Adults with irritable bowel syndrome reported no negative perceptions following hypnotherapy [29], consistent with our finding that stigma fades with early experience or information. A mixed-methods study on guided imagery also found that parents and children preferred psychological therapies over medications and had positive expectations [12]. Our study aligns with others showing varying willingness to engage with hypnotherapy, including scepticism and disbelief in its effectiveness [10], doctors being unaware of how to refer (or being unwilling to refer) [28, 29], and cost and time as barriers [11]. For example, a survey among GPs in the UK found varying willingness to recommend hypnotherapy for adults with irritable bowel syndrome and contrasting opinions about how hypnotherapy should be available (e.g. through accredited hypnotherapists or GPs) [31].

In our study, GPs mentioned a possible difficulty applying hypnotherapy in primary care because they see only a small group of children with functional abdominal pain. Evidence shows that only one in five children visit their GP in the year after a first episode of non-acute abdominal pain or diarrhoea [32], and that only some children return with abdominal pain or mental health problems [33]. However, the need for an evidence-based coping strategy may be high, with 50% of children still reporting that abdominal pain affected daily activities 1 year later [34].

Finally, it was interesting that several GPs in our study mentioned that hypnotherapy might be suitable for indications other than functional abdominal pain. Although hypnotherapy has proven efficacy in adults with functional abdominal pain [35, 36], studies with sufficient methodological quality are lacking in other patient groups, such as children with chronic fatigue, asthma, nausea, and headache [37, 38].

### Implications for research and practice

This study has shown varying views among children, parents, and GPs regarding the use of hypnotherapy for functional abdominal pain in primary care, with differing opinions on its acceptability and conditions necessary for successful implementation. Some GPs were willing to offer hypnotherapy themselves, and others rejected the approach and would not even refer patients. This highlights the need for GPs to overcome their hesitation, be well-informed about what hypnotherapy entails and the evidence underpinning its use, and have clear referral options when discussing it with patients. Face-to-face hypnotherapy, home-based guided hypnotherapy, or both may be appropriate. Future research must now study what implementation strategies are best suited to primary care. With proven effectiveness in this setting, hypnotherapy could be implemented as an early treatment strategy for children who visit their GP with functional abdominal pain.

## Conclusions

Children, parents, and GPs consider hypnotherapy as a suitable treatment for children with functional abdominal pain in primary care, but opinions on its acceptability vary. Perceptions of hypnotherapy are influenced by attitudes of children and parents to abdominal pain, therapeutic context, and societal constraints and considerations. Patients and GPs need clear information about hypnotherapy and its effects to overcome pre-existing stigma before further implementation is possible.

## Supplementary Material

cmaf066_Supplementary_Data

## Data Availability

The data underlying this article cannot be shared publicly due to privacy and ethical concerns. The data will be shared on reasonable request with the corresponding author.
