# Supplementary material for: Perceptions of hypnotherapy for children with functional abdominal pain: a qualitative study
Source: Fam Pract. 2025 Aug 26;42(5):cmaf066. doi: 10.1093/fampra/cmaf066 (PMC12411915; doi:10.1093/fampra/cmaf066)
Supplement: cmaf066_Supplementary_Data [file cmaf066_supplementary_data.zip › Supplemental_material.pdf]

Supplement 1. Interview guide

<u>**Children and parents**</u>

Opening question

- Could you tell me something about your/your child's abdominal pain?

Abdominal pain

- When you have/your child has abdominal pain, what do you/does your child do to reduce the pain?
- How do you feel about you/your child having abdominal pain?
- What do you/does your child notice in your daily life from the abdominal pain?

Healthcare

- How did your GP help you with the abdominal pain?
    - How did you feel about this?
- What did the GP explain about the complaints?
- What did the GP explain about treatment?

Hypnotherapy

- What is the first thing that comes to your mind when you hear the word 'hypnotherapy'?
- What do you know about hypnotherapy?
- Why is hypnotherapy important to you/your child?
- What do you hope that hypnotherapy can mean to you/your child?
- There is evidence that hypnotherapy reduces pain in children in paediatric care. How do you think this is possible?

Explanation is given about hypnotherapy: *I will give you more information about hypnotherapy and hypnosis. I am curious what you think of it afterwards. Hypnotherapy is more often used as medical hypnosis. During medical hypnosis, you are very relaxed and a therapist gives suggestions. This can be compared with daydreaming, where you forget your environment and use your own imagination. Suggestions from the therapist can have multiple aims, such as relaxation, or getting a calm belly. The suggestions are positive, and you are in charge, so you can think of fun stuff yourself.*

- Do you now have a clear image of hypnotherapy?
    - What else would you like to know?
- How did this information change your view on hypnotherapy?
- What advantage of hypnotherapy can you think of?
- What disadvantage of hypnotherapy can you think of?

Motivation

- Would you do/let your child do hypnotherapy?
    - Why (not)?
- What resists you to do hypnotherapy?
- What information do you need in advance?

Selfhypnosis

- Hypnotherapy can be performed at home. We call this selfhypnosis, where you can listen to hypnotherapy exercises which are audio-recorded by a therapist. What do you think about this?
    - Would you/let your child do selfhypnosis?
        - Why (not)?

Implementation

- Would you require help of someone, next to information on a website?
  - Of whom?
  - Why?
- When could hypnotherapy be offered to children with abdominal pain?
- How would you like to get there?
- *For parents:* How would you like to be involved?

Closing question

- What advice would you give other children/parents of children with abdominal pain?

__General practitioners__

Opening question

- Could you tell me something about your experience with children with functional abdominal pain or irritable bowel syndrome?

Policy

- What aim do you wish to achieve in the treatment of children with functional abdominal pain?
  - How?
- What is the GPs' role?
- Did you refer children to paediatric care?
  - Why?
  - How did you feel about this?

Hypnotherapy

- What is the first thing that comes to your mind when you hear the word 'hypnotherapy'?
- What do you know about hypnotherapy?
- There is evidence that hypnotherapy reduces pain in children in paediatric care. How do you think this is possible?

Explanation is given about hypnotherapy: *I will give you more information about hypnotherapy and hypnosis. I am curious what you think of it afterwards. Hypnotherapy is more often used as medical hypnosis. During medical hypnosis, you are very relaxed and a therapist gives suggestions. This can be compared with daydreaming, where you forget your environment and use your own imagination. Suggestions from the therapist can have multiple aims, such as relaxation, or getting a calm belly. The suggestions are positive, and you are in charge, so you can think of fun stuff yourself.*

- Do you now have a clear image of hypnotherapy?
  - What else would you like to know?
- How did this information change your view on hypnotherapy?

Hypnotherapy for abdominal pain

- How do you feel about hypnotherapy for children with functional abdominal pain and irritable bowel syndrome?
- What do you think that hypnotherapy can mean to children with functional abdominal pain?
- Did you ever consider hypnotherapy as treatment?
- How could hypnotherapy help to achieve the aim we discussed before?
- Why does hypnotherapy not help?
- What advantage of hypnotherapy can you think of for GPs?
  - And for patients?
- What disadvantage of hypnotherapy can you think of for GPs?
  - And for patients?

Selfhypnosis

- Hypnotherapy can be performed at home. We call this selfhypnosis, where patients can listen to hypnotherapy exercises which are audio-recorded by a therapist. What do you think about this?
    - Would you let your patients do selfhypnosis?
        - Why (not)?

Implementation

- Do you think that referral to a website including information sufficient?
    - Why (not)?
- Who would be the best person to give hypnotherapy?
    - Why?
- How would patients get there?
- When could GPs offer hypnotherapy to patients?
- What do you need to refer patients to be at ease?
- What resist you to implement hypnotherapy as treatment?
- What would facilitate implementation of hypnotherapy?

Closing question

What advice would you give other GPs concerning treatment of children with functional abdominal pain?

Supplement 2. Research team

Name	Gender	Background
I.N. Ganzevoort	Female	PhD candidate, MSc
A.L. Van der Veen	Female	Research assistant, MSc
M.A. Alma	Female	Social scientist with expertise in qualitative research, PhD
A. Karg	Female	General practitioner, MD
E. AB	Female	General practitioner, MD
M.Y. Berger	Female	Professor of general practice, general practitioner, epidemiologist, MD, PhD
G.A. Holtman	Female	Assistant professor, epidemiologist, PhD
